# RRM1 predicts clinical outcome of high-and intermediate-risk non-muscle-invasive bladder cancer patients treated with intravesical gemcitabine monotherapy

**DOI:** 10.1186/s12894-019-0497-x

**Published:** 2019-07-24

**Authors:** Zhenxing Yang, Bingqiang Fu, Luqiang Zhou, Jie Xu, Ping Hao, Zhenqiang Fang

**Affiliations:** 1 Department of Urology, Second Affiliated Hospital, Third Military Medical University, Chongqing, 400037 China; 2 SurExam Bio-Tech Co, Guangzhou, 510663 Guangdong China; 3 Department of Oncology, Second Affiliated Hospital, Third Military Medical University, Chongqing, 400037 China

**Keywords:** RRM1, Non-muscle-invasive bladder cancer, Gemcitabine

## Abstract

**Background:**

The expression level of ribonucleotide reductase subunit M1 (RRM1) is closely related to the effect of gemcitabine-based therapy in advanced bladder cancer. However, the value of RRM1 expression in predicting progression-free survival in non-muscle-invasive bladder cancer (NMIBC) patients treated with intravesical gemcitabine chemotherapy has not been elucidated.

**Methods:**

This study randomly assigned 162 patients to either the RRM1-known group or the unknown group. We collected cancer tissues from 81 patients to evaluate the mRNA expression of RRM1 by using liquid chip technology. All patients were diagnosed and then treated with intravesical gemcitabine monotherapy immediately after transurethral resection of the bladder tumour (TURBT).

**Results:**

RRM1 expression was high in 21% (17/81) of patients. The RRM1 mRNA level was not correlated with sex, age, weight, performance status, or CUA/EAU risk (*p* > 0.05). Progression-free survival (PFS) was significantly longer for patients with low RRM1 expression than for patients with high and unknown RRM1 expression (*p* = 0.009). Additionally, the 1- and 2-year relapse rates also differed according to RRM1 expression level. The 1-year relapse rates for RRM1-low, RRM1-high and RRM1-unknown patients were 0, 17.7 and 6.2% (*p* = 0.009), while the 2-year relapse rates for these groups were 3.1, 29.4, and 11.1% (*p* = 0.005), respectively.

**Conclusions:**

This preliminary study showed that low RRM1 expression was associated with longer progression-free survival and lower 1-year/2-year relapse rates in NMIBC patients treated with intravesical gemcitabine monotherapy, despite the need for further verification with large sample sizes and considering more mixed factors and biases.

## Background

Bladder tumours represent the ninth most prevalent malignancy in China, and they were responsible for an estimated 32,900 deaths in 2015 [1]. Approximately 70% of all bladder carcinomas are first diagnosed as non-muscle-invasive bladder cancer (NMIBC), including tumours of any grade at stages pTa, pT1, or carcinoma in situ (CIS) [2]. Unlike its muscle-invasive counterpart, NMIBC typically has a good prognosis. The EAU guidelines define NMIBC patients as low, intermediate, or high risk for recurrence (based upon stage, grade, tumour size, and multifocality) [3]. Patients with higher progression scores are more likely to progress to muscle invasion within 5 years. Transurethral resection of the bladder tumour (TURBT) is the diagnostic and gold standard treatment option for NMIBC. Despite visually complete resection, 30–80% of NMIBC patients will have disease recurrence, possibly due to invisible residual lesions or implantation of tumour cells during TURBT [4]. Current evidence suggests that subsequent instillations of intravesical chemotherapy are necessary for higher-risk disease [5]. Gemcitabine is a cell-cycle specific antimetabolite that is widely used in intravesical chemotherapy [6].

Ribonucleotide reductase subunit M1 (RRM1) is the largest catalytic subunit of ribonucleotide reductase (RR), which is the key enzyme catalysing the transformation of ribonucleoside diphosphates to deoxyribonucleoside diphosphates [7]. Gemcitabine is an analogue of deoxycytidine. The active forms of gemcitabine inhibit DNA synthesis by incorporating into the DNA chain or inhibiting RRM1 activity [8]. Gemcitabine has been widely used for the treatment of several aggressive solid tumour types, including non-small-cell lung cancer (NSCLC), bladder tumours, pancreatic tumours and nasopharyngeal carcinoma [9–12]. There are preclinical and clinical data indicating that high RRM1 protein levels in various cancers are associated with gemcitabine resistance [13, 14]. Moreover, several clinical studies have demonstrated the association between elevated RRM1 levels and unfavourable clinical outcomes in advanced bladder tumour patients treated with gemcitabine­based therapy [9, 15]. However, the relationship between RRM1 mRNA level and gemcitabine activity in NMIBC has not been addressed. In the current paper, we demonstrated the predictive and prognostic value of RRM1 in patients with NMIBC receiving intravesical gemcitabine chemotherapy.

## Methods

### Patients

This retrospective study enrolled 162 patients with histological confirmed NMIBC and intermediate/high-risk disease at the Second Affiliated Hospital of Third Military Medical University from November 2010 to January 2016. Tissue samples from patients were obtained after surgery. An Eastern Cooperative Oncology Group (ECOG) performance status (PS) of 0 to 2 was assessed in all enrolled patients. Patient inclusion criteria included the following: 1) an NMIBC patient diagnosis following the EAU guidelines; 2) intermediate or high-risk bladder cancer patients without lymph node metastasis or distant metastases; 3) all patients underwent transurethral resection of the bladder tumour plus subsequent instillations of intravesical gemcitabine chemotherapy; 4) first diagnosis of a bladder tumour without accepting any surgery or drug treatment; and 5) patients voluntarily participated in the study and signed the informed consent form. This study was conducted with the approval of the medical ethics committee of Second Affiliated Hospital of the Third Military Medical University. Each patient provided written informed consent before participation in the current investigation. Patient information on pathologic characteristics, treatment details, and survival was obtained from follow-up and surgical records. The major clinical endpoint in the current study was disease-free survival (DFS), and the secondary clinical outcomes were 1-year and 2-year relapse rates.

### Treatment and response evaluation

All patients received intravesical gemcitabine monotherapy immediately after TURBT. A total of 1000 mg of gemcitabine was diluted in 40 ml of saline solution, and patients received weekly instillations for 8 consecutive weeks. The drug was held in the bladder for 60 min. The treatment cycle was then changed to once a month for one year. Follow-up was performed to assess the efficacy of the treatment for all involved patients. In general, in the first year, cystoscopy and urinary cytology were examined at 3-month intervals and then at 6-month intervals in the next year. Relapse was defined as a positive examination on cystoscopy. The first recurrence (disease-free survival) time was defined as the period between TURBT and positive finding during cystoscopy.

### RRM1 mRNA expression analysis

In the current investigation, multiplex branched-DNA (bDNA) liquid chip technology was employed to perform RRM1 expression analyses at SurExam Medical Test Centre, Guangzhou, China, and these analyses are detailed in the paper published by Zhang [16]. Briefly, digested tissue was incubated with target gene-specific probe sets, and then fluorescence capture beads were added. Then, the mixture was hybridized with bDNA signal amplification probes for the purpose of signal amplification. In the end, the Luminex 200 system (Luminex Corp., Austin, Texas) was used to cluster the fluorescence value of each sample. Three standard genes were demonstrated as reference genes, including beta-2-microglobulin (B2M), transfer in receptor (TFRC), and TATA box-binding protein (TBP). All original data were subjected to standardize processing, which included raw data alignment (fastq file), duplication removal, quality control, etc., until we obtained clean data for subsequent analyses. All gene expression levels among patients were distributed across the whole samples, and then each patient received an RRM1 expression value. The median RRM1expression level was selected for the cutoff value. The expression level of RRM1 was considered high if its level was equal to or exceeded the cutoff value. All other mRNA values were considered low expression.

### Statistical analysis

SPSS statistical software, version 19.0 (IBM Corporation, Armonk, New York, USA) was employed for data analysis. The χ^2^ and Fisher exact tests were performed to identify associations between clinicopathologic variables and RRM1 status as appropriate. Survival curves were demonstrated by using the Kaplan-Meier method. A log-rank test was used to compare survival differences among groups. All statistical tests were two-sided, and a statistically significant difference was defined as *p* < 0.05.

## Results

### Patient features

Table 1 shows the baseline clinicopathologic features of the study population. The median age of the 162 patients was 60 years (range, 25–89 years), and there were 132 (81.5%) men and 30 (18.5%) women in the cohort. All patients were classified as having high-risk NMIBC and an ECOG PS 0~2. All patients underwent 1 year of intravesical gemcitabine chemotherapy after TURBT. The median patient follow-up was 30.5 months (range, 14–76 months). The patients were randomly assigned evenly to either the RRM1-known group (high and low expression of RRM1) or to the unknown group. There were 81 tumour samples successfully processed for RRM1 analysis, and 21.0% (17/81) of the patient demonstrated high RRM1 expression.

**Table 1:** Patients’ characteristics according to RRM1 expression

Features	*N*	RRM1 expression			λ^2^值	*p*
		Low (%)	High (%)	Unknown (%)		
Gender						
Female	30	11 (17.2)	3 (17.6)	16 (19.8)	0.166	0.921
Male	132	53 (82.8)	14 (82.4)	65 (80.2)		
Age (years)						
≤ 60	85	39 (60.2)	12 (70.6)	34 (42.0)	3.542	0.170
> 60	77	25 (39.8)	5 (29.4)	47 (58.0)		
Weight (Kg)						
≤ 65	78	26 (40.6)	8 (47.1)	44 (56.4)	2.695	0.260
> 65	84	38 (59.4)	9 (52.9)	37 (44.0)		
ECOG Score						
0–1	157	62 (96.9)	17 (100.0)	78 (96.3)	0.645	0.724
2	5	2 (3.1)	0	3 (3.7)		
CUA/EAU risk group						
2	54	22 (34.4)	6 (35.3)	26 (32.1)	0.116	0.944
3	108	42 (65.6)	11 (64.7)	55 (67.9)		
Relapse						
Yes	23	3 (4.9)	5 (29.4)	15 (18.5)	9.223	**0.010**
No	139	61 (95.1)	12 (70.6)	66 (81.5)		

*p* < 0.05 is set in boldface

### Relationship between clinicopathologic features and RRM1 expression

The expression of RRM1 was divided into high and low expression according to the cutoff value of RRM1, which was 0.557 (range 0.006–0.997). Of the 162 patients, RRM1 expression was high in 17 (10.5%) patients and low in 64 (39.5%) patients. The RRM1 level was unknown in the remaining 50% of patients. As shown in Table 2, the level of RRM1 expression was moderately associated with clinicopathologic features. There were no significant differences observed in sex (*p* = 0.921), age (*p* = 0.170), weight (*p* = 0.260), ECOG performance status (*p* = 0.610), or CUA/EAU risk between the three groups (*p* = 0.944). Surprisingly, when compared to patients with low (4.9%, 3/64) and unknown (18.5%, 15/81) RRM1 levels, patients with high RRM1 levels were more likely to relapse (29.4%, 5/17), *p* = 0.010.

**Table 2:** The 1-year/2-year relapse rates according to RRM1 expression and clinicopathological features

	No	1-y R^a^ rate (%)	*p*	2-y R^a^ rate (%)	*p*
RRM1 level					
Low	64	0	**0.009**	3.1%	**0.005**
High	17	17.7%		29.4%	
Unknown	81	6.2%		11.1%	
Gender					
Female	30	13.3%	**0.040**	13.3%	0.500
Male	132	3.0%		9.1%	
Age (years)					
≤ 60	85	4.7%	0.569	10.6%	0.75
> 60	77	5.3%		9.1%	
Weight (Kg)					
≤ 65	78	13.3%	0.204	7.7%	0.369
> 65	84	4.8%		11.9%	
ECOG Score					
0–1	157	5.1%	0.774	10.2%	0.452
2	5	0		0	
CUA/EAU risk group					
2	54	1.9%	0.271	11.1%	0.710
3	108	6.5%		9.3%	

*R*: means relapse

*p* < 0.05 is set in boldface

### Association between RRM1 level and clinical outcomes

**Fig. 1:**
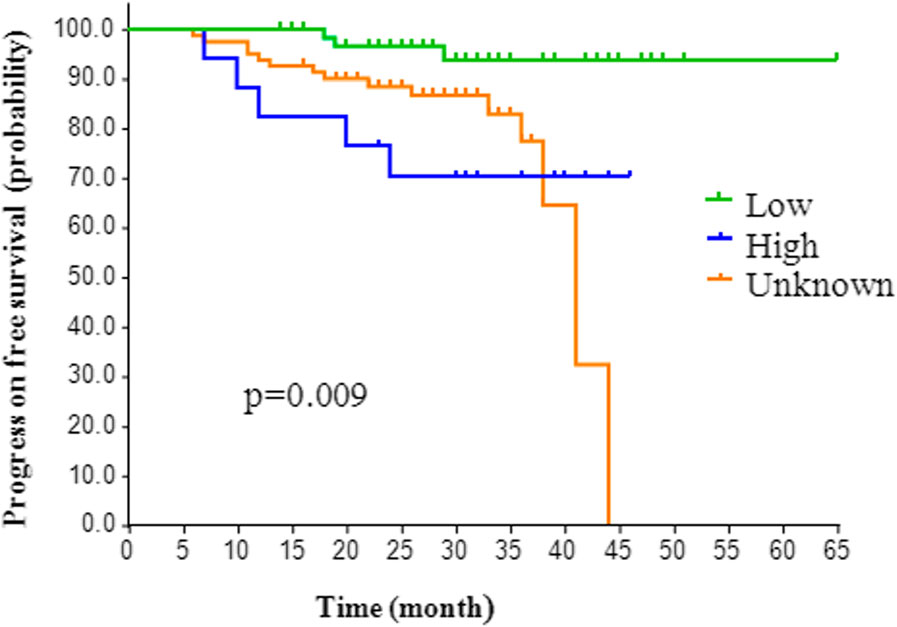
Analysis of the progression of disease-free survival probability among low, high and unknown groups

After a median follow-up of 30.5 months, 14.2% (23/162) of the patients experienced disease progression, and all patients were alive. As shown in Fig. 1, PFS was significantly longer for patients with low RRM1 expression than for patients with high and unknown RRM1 expression, *p* = 0.009. However, median PFS could not be calculated because a majority of patients were disease-free at the end of follow-up.

Additionally, the 1-year and 2-year relapse rates also differed according to RRM1 expression and other clinical characteristics. The 1-year relapse rates for RRM1-low, RRM1-high and RRM1-unknown patients were 0, 17.7 and 6.2% (*p* = 0.009), respectively, while the 2-year relapse rates in these groups were 3.1, 29.4, and 11.1% (*p* = 0.005), respectively. Other clinical characteristics, such as age, weight, and performance status, showed little association with 1-year/2-year relapse rates. The female patients had higher 1-year relapse rates than the male patients (13.3% vs. 3.0%, *p* = 0.04).

## Discussion

In clinical practice, the effects of chemotherapeutic agents or regimens vary among different individuals. Pharmacogenomics studies have shown that genetic factors play a vital role in curative effects. Therefore, identifying a biomarker is essential for establishing personalized treatments and improving treatment outcomes. Several recent studies have focused on evaluating predictive and/or prognostic markers for various tumours, including non-small-cell lung cancer (NSCLC), nasopharyngeal carcinoma and bladder carcinoma.

Our present study investigated the predictive and prognostic value of RRM1 in patients with NMIBC receiving intravesical gemcitabine chemotherapy. There were 162 NMIBC patients randomly assigned to either the RRM1-known group or the unknown group. The expression levels of RRM1 were significantly associated with age, sex, weight, ECOG performance status, or CUA/EAU risk, which is in accordance with prior results in advanced UC and NSCLC [9, 17]. Our data showed that high RRM1 expression was observed in less than 30% of tumours and was an unfavourable prognostic factor for PFS. Conversely, a low RRM1 level was found to be correlated with better PFS according to the Kaplan-Meier analysis. In addition, patients with low RRM1 expression showed the lowest 1-year/2-year relapse rate, while the RRM1-high patients had the highest relapse rates (17.1 and 29.4%, respectively). The association between RRM1 expression and prognosis in the present study was consistent with previous studies investigating other tumours treated with gemcitabine.

Previous studies associated with RRM1 expression in NSCLC patients largely demonstrated that low or negative RRM1 levels in patients with advanced NSCLC receiving gemcitabine-based regimens were correlated with higher response rates and a better prognosis [18]. In addition, there are also several studies that evaluated the predictive and/or prognostic value of RRM1 expression level in patients with urothelial carcinoma (UC). In one study, high RRM1 expression in respectable MIBC patients aged < 70 years was associated with improved survival [19]. On the other hand, the RRM1 level in advanced BC patients receiving gemcitabine-based regimes was not correlated with response or OS, except time-to-progression (TTP) [20]. These discrepant results may be attributable to differences in the patients involved. Similar to our results, Kim et al. found that high RRM1 expression was associated with inferior prognosis and clinical outcome after platinum plus gemcitabine combination chemotherapy for advanced UC [9, 15]. In contrast with previous studies regarding RRM1 in UC with gemcitabine-based therapy, this is the first study of early BC (NMIBC) with intravesical gemcitabine monotherapy. Taken together, these studies suggest that low RRM1 expression may help identify patients who will significantly benefit from gemcitabine-based chemotherapy in early and advanced UC.

The role of RRM1 in gemcitabine resistance carries more significance in the MIBC population, in which gemcitabine chemotherapy in combination with cisplatin is the standard of care. The standard treatment of NMIBC, however, remains treatment with bacillus Calmette–Guerin (BCG). The role of gemcitabine as a standard intravesical treatment in non-BCG refractory patients is unclear, as recurrence rates have been shown to be significantly higher among high-risk patients treated with gemcitabine compared with those treated with BCG. BCG was the first choice in intravesical instillation treatment with intermediate- or high-risk NMIBC. The recurrence rate of BCG treatment is lower than that of gemcitabine intravesical chemotherapy. However, in China, the price of BCG is too high, approximately 6 times higher than the cost of gemcitabine treatment, and no medical insurance covers this cost. For economic reasons, most NMIBC patients generally give up using BCG treatment and use gemcitabine intravesical chemotherapy as a secondary choice. The purpose of current study was to maximize the efficacy of gemcitabine perfusion therapy in these patients.

Nevertheless, limitations must be considered in the current investigation. The current study is a single-centre study, and the number of RRM1-high patients investigated was relatively small (*N* = 17). In addition, the Kaplan-Meier analysis of RRM1-low and RRM1-unknown groups failed to reach median PFS despite having a median follow-up of 30.5 months (range, 14–76 months). One main reason for these limitations is that the prognosis of NMIBC patients is usually good. Therefore, further well-designed studies must enrol a larger sample size and have longer follow-up periods. In addition, the nature of the retrospective study means that there is a risk of selection bias, which is another limitation in our investigation, and a lack of secondary analysis (regression models) to confirm RRM1 as an independent variable associated with disease recurrence and progression should be avoided in future studies. Despite these limitations, the current study is clinically meaningful and suggests the important role of RRM1 mRNA expression in patients with NMIBC. To our knowledge, this is the first study evaluating RRM1 mRNA in NMIBC patients treated with intravesical gemcitabine monotherapy.

## Conclusions

In conclusion, this preliminary study showed that low RRM1 expression was associated with longer progression-free survival and lower 1-year/2-year relapse rates in NMIBC patients treated with intravesical gemcitabine monotherapy, despite the need for further verification with large sample sizes and considering more mixed factors and biases.

## Data Availability

The datasets for the current study are available from the corresponding author upon reasonable request.

## Abbreviations

CUA: China Urological Association
EAU: European Association of Urology
NMIBC: Non-muscle-invasive bladder cancer
PFS: Progression-free survival
RRM1: Ribonucleotide reductase subunit M1
TURBT: Transurethral resection of the bladder tumour
